# Evaluation of eight reference genes for quantitative polymerase chain reaction analysis in human T lymphocytes co-cultured with mesenchymal stem cells

**DOI:** 10.3892/mmr.2021.11920

**Published:** 2021-02-17

**Authors:** Xiuying Li, Qiwei Yang, Jinping Bai, Yali Xuan, Yimin Wang

Mol Med Rep 12: 7721-7727, 2015; DOI: 10.3892/mmr.2015.4396

Following the publication of the above article, the authors contacted the Editorial Office to explain that Fig. 1A and some of the images in Fig. 1B in the paper had already been published in Fig. 1 in another article by the same authors, and they had forgotten to cite the former publication. The paper in which these data appeared was as follows: Li X, Yang Q, Bai J, Xuan Y and Wang Y: Identification of appropriate reference genes for human mesenchymal stem cell analysis by quantitative real-time PCR. Biotechnol Lett 37: 67–73, 2015.

Fig. 1 of the above paper is reprinted opposite, now with the original source of the figure acknowledged in the form of a reference citation at the end of the Figure caption. The authors apologize to the publishers of *Biotechnology Letters* for having failed to include a proper acknowledgement for use of the figure in the above publication.

## Figures and Tables

**Figure 1.**
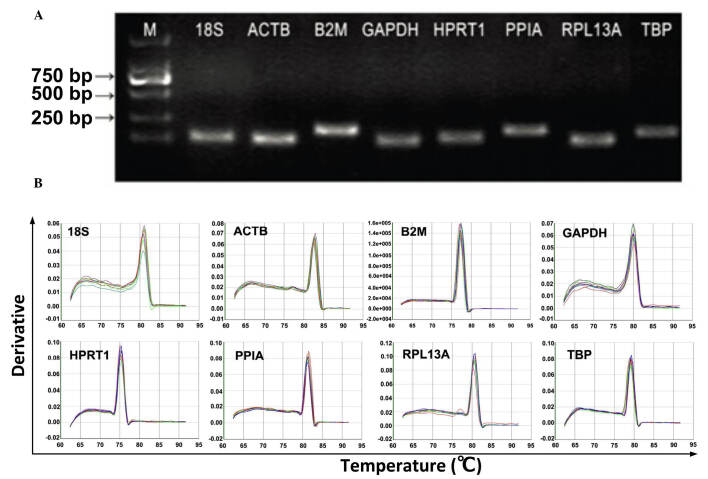
Primer specificity and amplicon length. The PCR amplification products were analyzed using agarose gel electrophoresis and dissociation curves. (A) PCR products were run on a 2% agarose gel. The presence of a single band with anticipate size indicated the PCR product was specific (B) Dissociation curves for the PCR products. The single peak indicates a specific PCR product. PCR, polymerase chain reaction; M, marker; 18S, 18S ribosomal RNA; PPIA, peptidyl-prolylisomerase A; RPL13A, ribosomal protein L13a; HPRT1, hypoxanthinephosphoribosyl transferase 1; ACTB, β-actin; B2M, β-2-microglobulin; GADPH, glyceraldehyde-3-phosphate dehydrogenase; TBP, TATA box-binding protein. Note that Fig. 1A, and the images for ACTB, HPRT1 and TBP in Fig. 1B, were taken from the following paper: Li X, Yang Q, Bai J, *et al*. Identification of appropriate reference genes for human mesenchymal stem cell analysis by quantitative real-time PCR. Biotechnol Lett 37: 67–73, 2015. https://doi.org/10.1007/s10529-014-1652-9.

